# An intraoperative device to restore femoral offset in total hip arthroplasty

**DOI:** 10.1186/s13018-014-0058-7

**Published:** 2014-07-19

**Authors:** Enxing Xue, Zhen Su, Chengwang Chen, Paul Kim Chiu Wong, Hong Wen, Yu Zhang

**Affiliations:** 1 Department of Orthopaedic Surgery, The Second Affiliated Hospital and Yuying Children’s Hospital of Wenzhou Medical University, 109 Xueyuan West Road, Wenzhou 325000, Zhejiang, China; 2 Department of Nephrology, The First Affiliated Hospital of Wenzhou Medical University, Wenzhou 325000, Zhejiang, China; 3 Department of Surgery, University of Toronto, 650 Sammon Ave. Suite 301, Toronto M4C 5M5, ON, Canada

**Keywords:** Leg length discrepancy, Total hip arthroplasty, Femoral offset, Device

## Abstract

**Background:**

Leg length discrepancy (LLD) after total hip arthroplasty (THA) can lead to unsatisfactory outcome. Our objective was to design and evaluate a simple and reliable intraoperative device (Length-offset Lever) to minimize leg length discrepancy.

**Methods:**

This device was used in 51 patients undergoing primary total hip replacements. The leg length discrepancy was measured pre- and postoperatively based on plain radiographs.

**Results:**

Preoperative radiographic leg length discrepancy averaged 13.5 ± 6.2 mm. Leg length discrepancy showed significant improvement, with a postoperative average of 4.1 ± 2.3 mm (*p* < 0.0001). There were no complications associated with this device.

**Conclusions:**

The ‘Length-offset Lever’ is a useful intraoperative tool to restore anatomic femoral offset and height of femoral head.

## Introduction

One of the main goals of total hip arthroplasty is to restore and optimize normal hip biomechanics. Femoral offset is defined as the perpendicular distance between the centre of femoral head and the long axis of the femoral component. Charnley [1] has highlighted the importance of femoral offset on the hip joint forces.

Leg length discrepancy (LLD) after total hip arthroplasty (THA) is one of the leading causes for unsatisfactory outcomes [2]–[7]. Various methods have been described to measure LLD directly or indirectly, during THA [8]–[11].

**Figure 1:**
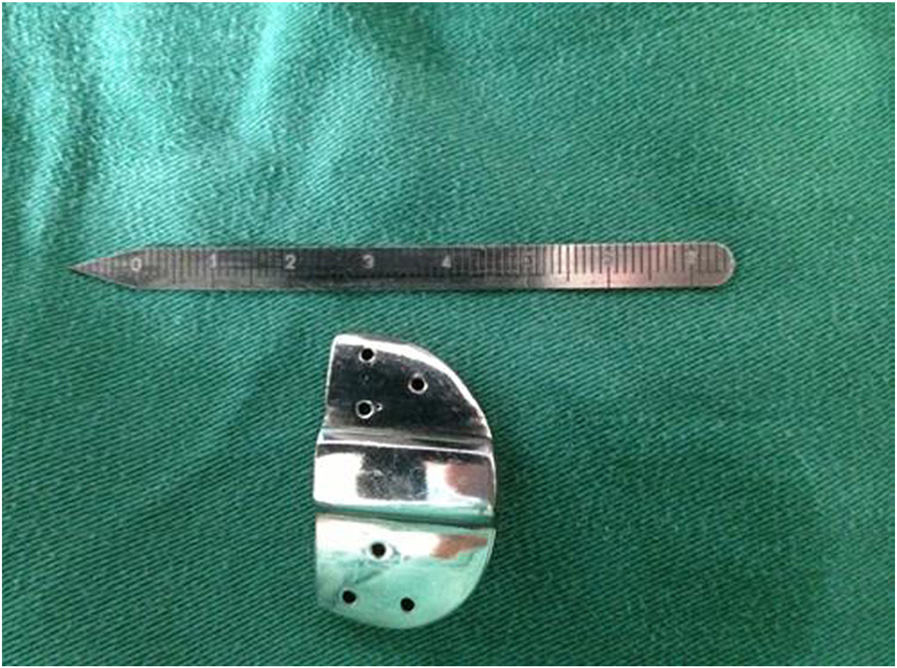
Length-offset Lever consists of a base and a measuring ruler.

This paper describes a simple, reliable and safe intraoperative device, Length-offset Lever. It can be used to perform THA, done through a direct lateral approach, without requiring additional equipment, incisions, or radiographs (Figure 1). It is a ‘two-in-one’ device used to measure femoral offset, intraoperatively.

## Methods

### Patients

In our study, preoperative diagnoses were Crowe I or II developmental dysplasia of the hip in 23 patients, osteonecrosis in 12, osteoarthritis in 10, and rheumatoid arthritis in 6. Of all the patients, 22 were male and 29 were female. This study was approved by the ethics committee of The Second Affiliated Hospital of Wenzhou Medical University, and informed consents were obtained from participants or their authorized persons.

We studied a total of 51 patients undergoing primary THA between March 2008 and December 2011, using the Length-offset Lever. Revisions, THAs requiring osteotomies, and first operations of staged bilateral THAs were excluded. All THAs in this study were performed with cementless fixation utilizing direct lateral approach. Synergy stems (Smith & Nephew, Andover, MA, USA) were used in 28 cases, Tri-lock and Corail stems (Depuy, Warsaw, IN, USA) in 19 cases, and M/L stems (Zimmer, Warsaw, IN, USA) in 4 cases.

### Surgical technique

**Figure 2:**
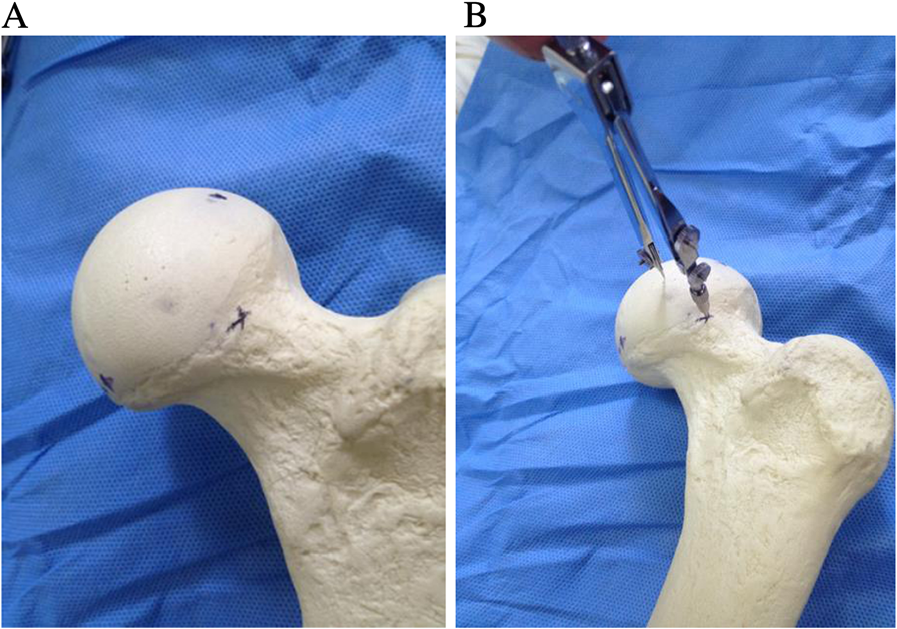
**The method to determine the center of the femoral head. (A)** Three points on the boundary of femoral head. **(B)** The center is equidistant to three points by compasses.

**Figure 3:**
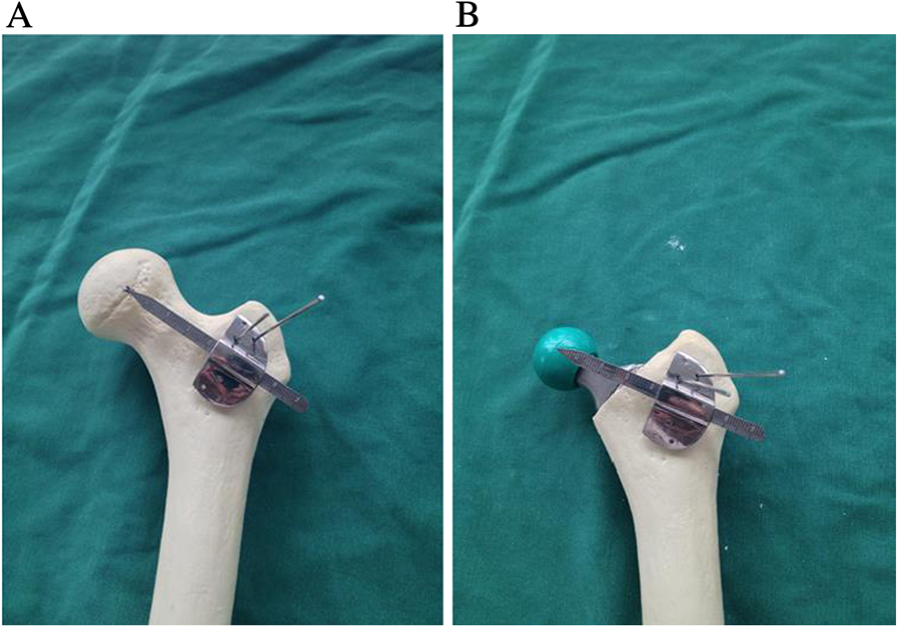
**Length-offset Lever for THA. (A)** Femoral offset measurement using ‘Length-offset Lever’ before femoral preparation. **(B)** Femoral offset measurement with trial components.

Fifty-one patients were placed in the lateral position and direct lateral approach to the hip was used. Length-offset Lever consisted of a ‘base’ and ‘measuring ruler’. After dislocation of the hip and prior to the femoral neck cut, we determined the center of the femoral head with the following procedures: first, we let compasses be perpendicular to the plane of the femoral head; then we find three points on the boundary of the femoral head and the center is equidistant to three points by compasses (Figure 2), the ‘base’ unit was fixed to the anterior aspect of the greater trochanter using K-wires. The anatomic femoral offset was measured after sliding the ruler's tip to the centre of the femoral head (Figure 3A). The ‘ruler’ was then removed, leaving the ‘base’ alone. After inserting the trial components, the ‘ruler’ was re-inserted into the ‘base’, and the new offset of the femoral head was then measured (Figure 3B). If we planned to lengthen the leg by 10 mm, we can have the femoral head trial 10 mm above the tip of the ruler. The fovea should be cleared of soft tissue to evaluate acetabular depth, and transverse acetabular ligament can be used to confirm orientation and height of the acetabular component.

### Postoperative management

**Figure 4:**
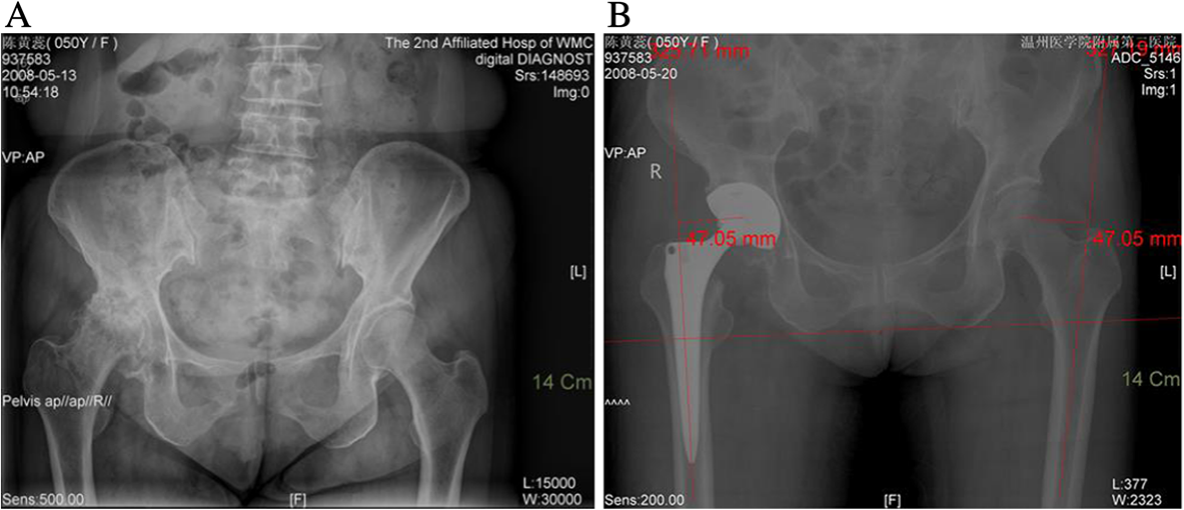
**Radiographs of pelvis of a 50-year-old female.** Presented with severe pain and limited range of motion in her right hip, secondary to osteoarthritis. **(A)** Preoperative limb-length inequality was 14.6 mm. **(B)** THA was performed using the Length-offset Lever (Figure 1). Postoperative limb-length inequality was 0 mm. The patient had excellent pain relief and functional results following the THA and no complications.

Leg length discrepancy was evaluated from an anteroposterior radiograph of the pelvis with both hips in neutral position (Figure 4). Radiographs were recorded on PACS (Picture Archiving & Communication System, INFINITT Co., Ltd, Guro-gu, Seoul, Korea) with a resolution of 1,536 × 2,048 pixels. A horizontal line was drawn through the bottom of the ischial tuberosities. Discrepancy in leg length was measured using the relationship between this line and the most prominent point of the lesser trochanter.

Clinical evaluations were based on the Harris hip scores [12]. Student's *t* tests were performed to determine statistical significance.

The postoperative average of femoral offset compared to the contralateral normal one on radiographs was recorded on PACS. Statistical analysis of the measurements was conducted using paired *t* tests, setting the level of significance at 0.05.

## Results

Mean age of patients was 65.59 years (range 18 to 84). The average follow-up period was 18 months (range 12 to 30). Preoperative radiographic leg length inequality averaged 13.5 ± 6.2 mm (range +33 to −12 mm). Leg length discrepancy showed significant improvement, with a postoperative average of 4.1 ± 2.3 mm (range 0 to 5) (*p* < 0.0001). None of the patients required shoe lifts for equalization of leg lengths nor complained of leg length discrepancy, postoperatively. No complications associated with Length-offset Lever were observed in these patients. There were no complications of dislocation, infection, fracture, or sciatic nerve palsy.

Harris hip scores significantly improved postoperatively. They increased from 45.9 ± 14.2 points preoperatively to 83.3 ± 12.6 points at the latest follow-up (*p* < 0.0001).

The paired *t* test comparisons showed statistically no significance difference between postoperative average of femoral offset and the contralateral normal one (*p* > 0.50).

## Discussion

It can be a difficult task to maintain hip stability while trying to achieve equal leg length during THA [13]–[17]. A longer neck length is often used to improve the stability of THA. With modern femoral component designs, surgeons have more options to balance the leg length while maintaining hip stability. One example is the modular femoral neck component, which allows for flexible adjustment of femoral offset and version.

Konyves and Bannister [2] found that patients with lengthened operative legs had poorer Oxford hip scores than those that were shorter or had equal leg lengths. They believed that LLD was most commonly caused by over-lengthening of the prosthetic head-neck distance. The accuracy of femoral offset measurement using preoperative plain radiographs is affected by the rotation of the femur and pelvic tilt [18],[19]. Surgeons, in the past, have used methods or devices like pins, rulers, and calipers for intraoperative measurement of leg length discrepancy [8],[10],[20],[21]. It can be employed easily for intraoperative limb length measurement during THA, without creating a separate incision. The average leg length discrepancy postoperative was 4.2 mm in our study. No complications associated with the use of this device were observed. None of the patients expressed dissatisfaction about limb-length inequality after surgery. Safety and effectiveness of the Length-offset Lever was demonstrated in our study.

The device is effective when the leg length discrepancy is secondary to femoral deformity with minimal acetabular bone loss. Its use may not be appropriate for cases with significant acetabular dysplasia and this device does not measure the overall leg length or global offset.

There are many methods to restore femoral offset to eliminate the leg length discrepancy. For example, preoperative templating is mentioned as a common method for many surgeons which is an indirect method, its result is affected by radiographic magnification and position of patients when they had X-ray, templating overlays enlarged by a uniform factor may not be perfectly accurate. The shuck test and the dropkick test both rely on soft tissue tension as a surrogate indicator of limb length, motor blockade from spinal or epidural anesthesia may cause them to be less reliable than standard general anesthesia [22]. Surgeons also should not rely solely on the intraoperative leg-to-leg comparison because it is limited by greater adduction on the operative side and inaccuracy of palpation of landmarks through surgical drapes. Another method [23] was performed with procedures of stitching a suture into the skin superior to the surgical field and drilling a pin into the ilium bicortically, superior to the acetabulum and perpendicular to the table. It had the drawbacks of the potential loosening of the pin and positional variation of the hip during measurement. Our tool has many advantages, such as ease of use, low cost, unaffected by soft tissue tension, and measurement of the femoral offset directly and intraoperatively.

In conclusion, the Length-offset Lever is a useful intraoperative tool to restore anatomic femoral offset and height of femoral head.

## Abbreviations

LLD: leg length discrepancy

THA: total hip arthroplasty

## Competing interests

The authors declare that they have no competing interests.

## Authors' contributions

EX and ZS contributed equally to this paper. Both wrote the manuscript, participated in the design of the study and performed the statistical analysis. YZ was involved in the statistical analysis, preparation of the figures, data interpretation, study design, and writing of the manuscript. PKCW was involved in the data interpretation and writing of the manuscript. CC and HW were involved in the review of published work and data interpretation. All authors read and approved the final manuscript.

## References

[B1] Charnley J Low Friction Arthroplasty of the Hip: Theory and Practice 1979 Springer-Verlag, New York

[B2] Konyves A Bannister GC The importance of leg length discrepancy after total hip arthroplasty J Bone Joint Surg (Br) 2005 87 155 10.1302/0301-620X.87B2.14878 15736733

[B3] Yamaguchi T Naito M Aasayama I Ishiko T Total hip arthroplasty: the relationship between posterolateral reconstruction, abductor muscle strength, and femoral offset J Orthop Surg (Hong Kong) 2004 12 164 15621900 10.1177/230949900401200205

[B4] McGrory BJ Morrey BF Cahalan TD An KN Cabanela ME Effect of femoral offset on range of motion and abductor muscle strength after total hip arthroplasty J Bone Joint Surg (Br) 1995 77-B 865 7593096

[B5] Asayama I Naito M Fujisawa M Kambe T Relationship between radiographic measurements of reconstructed hip joint position and the Trendelenburg sign J Arthroplasty 2002 17 747 10.1054/arth.2002.33552 12216029

[B6] Asayama I Chammongkich S Simpson KJ Kinsey TL Mahoney OM Reconstructed hip joint position and abductor muscle strength after total hip arthroplasty J Arthroplasty 2005 20 414 10.1016/j.arth.2004.01.016 16124955

[B7] Sakalkale DP Sharkey PF Eng K Hozack WJ Rothman RH Effect of femoral component offset on polyethylene wear in total hip arthroplasty Clin Orthop 2001 388 125 10.1097/00003086-200107000-00019 11451111

[B8] Naito M Ogata K Asayama I Intraoperative limb length measurement in total hip arthroplasty Int Orthop 1999 23 31 10.1007/s002640050298 10192014 PMC3619784

[B9] Ranawat CS Rajesh R Rodriquez JA Bhende HS Correction of limb length inequality during total hip arthroplasty J Arthroplasty 2001 16 715 10.1054/arth.2001.24442 11547369

[B10] Shiramizu K Naito M Shitama T Nakamura Y Shitama H L-shaped caliper for limb length measurement during total hip arthroplasty J Bone Joint Surg (Br) 2004 86 966 10.1302/0301-620X.86B7.14587 15446519

[B11] Takigami I Itokazu M Itoh Y Matsumoto K Yamamoto T Shimizu K Limb-length measurement in total hip arthroplasty using a calipers dual pin retractor Bull NYU Hosp Jt Dis 2008 66 2 107 18537779

[B12] Harris VM Traumatic arthritis of the hip after dislocation and acetabular fractures: treatment by mold arthroplasty. An end-result study using a new method of result evaluation J Bone Joint Surg Am 1969 51 737 5783851

[B13] Kelley SS High hip center in revision arthroplasty J Arthroplasty 1994 9 503 10.1016/0883-5403(94)90097-3 7807108

[B14] Charles MN Bourne RB Davey JR Greenwald AS Morrey BF Rorabeck CH Soft-tissue balancing of the hip: the role of femoral offset restoration Instr Course Lect 2005 54 131 15948440

[B15] Bourne RB Rorabeck CH Patterson JJ Guerin J Tapered titanium cementless total hip replacements: a 10- to 13-year follow-up study Clin Orthop 2001 393 112 10.1097/00003086-200112000-00013 11764339

[B16] Konyves A Bannister GC The importance of leg length discrepancy after total hiparthroplasty J Bone Joint Surg (Br) 2005 87-B 155 10.1302/0301-620X.87B2.14878 15736733

[B17] Steinberg B Harris WH The offset problem in total hip arthroplasty Contemp Orthop 1992 24 556

[B18] Plaass C Clauss M Ochsner PE Ilchmann T Influence of leg length discrepancy on clinical results after total hip arthroplasty: a prospective clinical trial Hip Int 2011 21 441 10.5301/HIP.2011.8575 21818744

[B19] Tannast M Zheng G Anderegg C Burckhardt K Langlotz F Ganz R Siebenrock KA Tilt and rotation correction of acetabular version on pelvic radiographs Clin Orthop Relat Res 2005 438 182 10.1097/01.blo.0000167669.26068.c5 16131889

[B20] McGee HMJ Scott JHS A simple method of obtaining equal leg length in total hip arthroplasty Clin Orthop Relat Res 1985 194 207 3978924

[B21] Itokazu M Masuda K Ohno T Itoh Y Takatsu T Wenyi Y A simple method of intraoperative limb length measurement in total hip arthroplasty Bull Hosp Jt Dis 1997 56 204 9438079

[B22] Sathappan SS Ginat D Patel V Walsh M Jaffe WL Di Cesare PE Effect of anesthesia type on limb length discrepancy after total hip arthroplasty J Arthroplasty 2008 23 2 203 209 10.1016/j.arth.2007.01.022 18280413

[B23] Ng VY Kean JR Glassman AH Limb-length discrepancy after hip arthroplasty J Bone Joint Surg Am 2013 95 15 1426 1436 10.2106/JBJS.L.00433 23925749

